# A Microfluidic System for Stable and Continuous EEG Monitoring from Multiple Larval Zebrafish

**DOI:** 10.3390/s20205903

**Published:** 2020-10-19

**Authors:** Yuhyun Lee, Hee Won Seo, Kyeong Jae Lee, Jae-Won Jang, Sohee Kim

**Affiliations:** Department of Robotics Engineering, Daegu Gyeongbuk Institute of Science and Technology (DGIST), Daegu 333, Korea; 0645753@naver.com (Y.L.); shw198@dgist.ac.kr (H.W.S.); kj.lee@dgist.ac.kr (K.J.L.); cjw944@dgist.ac.kr (J.-W.J.)

**Keywords:** larval zebrafish, electroencephalogram (EEG), microfluidic channel, agarose-free, drug screening, anti-epileptic drugs

## Abstract

Along with the increasing popularity of larval zebrafish as an experimental animal in the fields of drug screening, neuroscience, genetics, and developmental biology, the need for tools to deal with multiple larvae has emerged. Microfluidic channels have been employed to handle multiple larvae simultaneously, even for sensing electroencephalogram (EEG). In this study, we developed a microfluidic chip capable of uniform and continuous drug infusion across all microfluidic channels during EEG recording. Owing to the modular design of the microfluidic channels, the number of animals under investigation can be easily increased. Using the optimized design of the microfluidic chip, liquids could be exchanged uniformly across all channels without physically affecting the larvae contained in the channels, which assured a stable environment maintained all the time during EEG recording, by eliminating environmental artifacts and leaving only biological effects to be seen. To demonstrate the usefulness of the developed system in drug screening, we continuously measured EEG from four larvae without and with pentylenetetrazole application, up to 60 min. In addition, we recorded EEG from valproic acid (VPA)-treated zebrafish and demonstrated the suppression of seizure by VPA. The developed microfluidic system could contribute to the mass screening of EEG for drug development to treat neurological disorders such as epilepsy in a short time, owing to its handy size, cheap fabrication cost, and the guaranteed uniform drug infusion across all channels with no environmentally induced artifacts.

## 1. Introduction

Larval zebrafish have been used as an experimental animal in genetics, developmental biology, neuroscience, and social behavior due to many advantages: small-size, mass reproduction, transparent body, short development time after fertilization, and similar disease genetics with human by about 70% [1,2,3,4,5]. Its economic advantage such as handy size and mass reproduction can maximize the time and cost efficiency in experiments if tools to treat a lot of larvae at once are available.

Owing to such advantages, larval zebrafish have also been used in drug screening research. One of the target diseases is epilepsy, which is a neurological disorder accompanying irregular seizure [6,7,8,9]. Behavioral observation, target protein detection (e.g., c-Fos), and cell imaging have been common methods using larvae for screening anti-epileptic drugs (AEDs) [10,11,12,13]. On the other hand, recording electroencephalogram (EEG) is an established method to diagnose epilepsy in clinics, with its characteristic signals. Baraban et al. (2005) reported a method to record EEG from a single larva embedded in agarose [11]. Using the technique, EEG signals from various epileptic models induced by drugs, hyperthermia, and genetic modification could be measured [10,14,15,16,17]. EEG signals from epileptic larval zebrafish embedded in agarose have been reported to be similar to signals from mouse, rat, and human with epilepsy [18]. However, using agarose to immobilize the larva has several limitations since agarose adversely affects the survival and development of larvae [19,20] as well as the washing out of previous media is difficult [21]. In addition, the use of agarose as a delivery medium may cause delayed and unbalanced drug delivery. To improve the survival rate by eliminating the use of agarose, Meyer et al. (2016) used non-invasive EEG electrodes in a microarray, but they also used a single larva at once [20]. Hong et al. (2016) devised a method to record EEG from multiple larvae using microfluidic channels, without using agarose, and recorded EEG signals non-invasively using the electrodes fabricated inside the microfluidic channels [21]. Although zebrafish were not hurt during recording in their system, degradation of signal quality might occur due to the movement of fluid and fish, or poor contact between the fish and the electrode in the channel. On the other hand, Ball et al. (2009) proposed the advantage of invasive recording over non-invasive methods, as higher signal-to-noise ratios (SNRs) can be achieved by removing artifacts, including movements of the body or the eyes in human [22]. Because the zebrafish also moves its gills, eyes, or tail, invasive methods may be able to minimize such artifacts [9,21]. Recently, Eimon et al. (2018) tried to measure epileptic signals invasively from multiple larvae by fixing them in agarose, suggesting the continued need for researchers to record EEG from multiple larval zebrafish [23].

Research utilizing zebrafish has been expedited by adapting the microfluidics technology [5,24,25]. Microfluidic channels have been applied to treat larval zebrafish in drug treatment [19,26], developmental biology [26], cardiology [27], brain activity mapping [28], EEG [21], and electromyography [24] studies. They can align and trap multiple larvae in parallel in the liquid-filled environment. Zebrafish, an aquatic animal, has a fatal limitation over rodents in that it requires water during the whole research procedure including handling, surgery, and experiment [29]. During the experiment, microfluidic channels need to be filled with liquid so that zebrafish can be controlled through the fluid in the channel. By means of such microfluidic systems, physiological characteristics of zebrafish in water can be preserved and recorded using sensors located inside or put into the channels from outside.

Many approaches using microfluidic channels to sense electrophysiological signals have adapted ladder-shaped fluidic channels [27,28]. Those devices have an inlet, an outlet, and multiple parallel trapping channels between them, which are arranged perpendicular to the pathway from the inlet to the outlet. However, trapping zebrafish in such channels is challenging, due to uncontrolled spontaneous swimming of zebrafish and pressure differences caused by zebrafish blocking the flow pathway. To overcome such technical limitations, Cho et al. (2020) proposed a design in which outlets were connected with trapping channels one by one [24]. In addition, similar designs of microfluidic channels have been developed in imaging and monitoring the embryonic development according to drug treatment [19,26]. Bischel et al. (2013) used branch-shaped microfluidic channels to place zebrafish embryos in a predictable way [19]. They added drugs through access ports at the locations where zebrafish were placed. Additionally, Choudhury et al. (2012) used the microfluidic channels with increasing numbers of branches toward the outlets, to investigate the concentration gradient of mixture when two different drugs were infused through two inlets [26].

In the present study, we proposed a system to record EEG invasively from multiple larval zebrafish using optimized microfluidic channels, in which the location of fish could be controlled precisely without using agarose at any experimental steps. In previous studies employing invasive EEG [10,11,23], agarose was used to fix the zebrafish in place accurately, while placing the needle electrode onto the zebrafish’s head and to infuse drugs, although the aforementioned shortcomings of agarose were pointed out. The proposed microfluidic channels were capable of uniform and continuous drug infusion without delays across the channels to observe the changes in EEG signals sequentially by each drug, while keeping individual fish unaffected by environmental changes. In many cases, embryo medium or anesthetic agents are used first to record default EEG (i.e., baseline) and then, drugs of interest, such as pentylenetetrazole (PTZ) or AEDs, are applied to the same fish sequentially [11,20,21,30,31]. Thus, the capability of microfluidic channels to keep zebrafish in their desired place stably during the whole experimental section and to sequentially exchange liquids with little effects on the zebrafish contained in the channel must be guaranteed for mass drug screening applications. Thereby, any environmental artifacts in EEG can be eliminated, leaving only biological effects of drugs to be detected. Finally, we successfully analyzed the obtained EEG signals quantitatively, demonstrating the distinctive effects of convulsant and AED.

## 2. Methods

### 2.1. Design and Fabrication of a Microfluidic Chip

**Figure 1:**
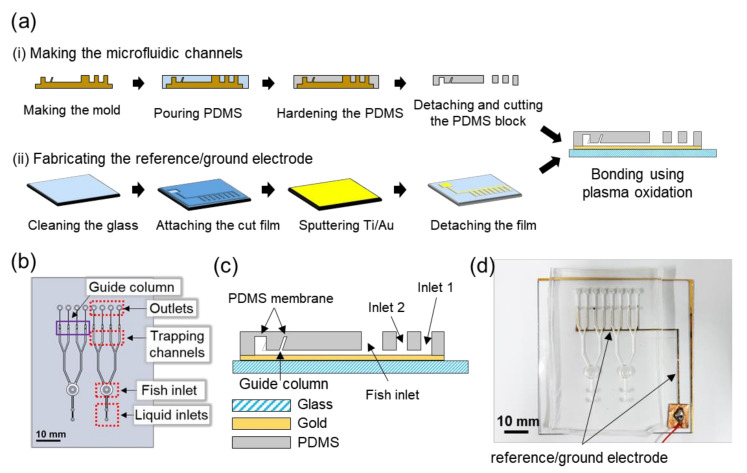
Design and fabrication of the microfluidic chip. (**a**) Fabrication process of (i) the microfluidic channels patterned in a PDMS block and (ii) the reference/ground electrode sputtered on a glass substrate. The structured PDMS block and the glass substrate were bonded together using plasma treatment. Schematics of the microfluidic chip containing microfluidic channels from (**b**) the top and (**c**) side views. (**d**) Photograph of the completed microfluidic chip containing gold patterns of reference/ground electrode at the bottom of the channels.

Figure 1a shows the fabrication process of the microfluidic chip to trap zebrafish during EEG recording. The mold of the designed microfluidic chip was printed using a 3D printer (Projet 3500, 3D Printers, Rock Hill, SC, USA). Parylene was coated (OBTPC200, OBANG technology, Gimpo-Si, Korea) on the front and back sides of the mold with a thickness of 1 to 3 μm, to facilitate easy separation of the fabricated chip and the mold. Polydimethylsiloxane (PDMS) (HeeSung STS, Seoul, Korea) mixed with curing agent at a weight ratio of 10:1 was poured onto the mold and hardened in an oven at 65 °C for 4 h. When PDMS was hardened, it was peeled off gently.

In the designed microfluidic chip, the reference/ground electrode was located at the bottom of each trapping channel, in a dimension of 0.5 mm × 5.44 mm. Using a film cutter (CE5000-40-CRP, Graphtec Corp., Yokohama, Japan), an adhesive film was cut into designed shape as a mask and placed onto a glass substrate cleaned using acetone, isopropyl alcohol, and distilled water. Titanium/gold with thicknesses of 50/200 nm were sputtered (DKC161125, Daeki Hi-Tech, Daejeon, Korea) on the glass. The structured PDMS block and the gold-patterned glass were treated with oxygen plasma (CUTE-MPR, Femto Science Co., Hwaseong-Si, Korea) and bonded to each other by aligning the trapping channels and the gold ground electrodes. The completed microfluidic chip is shown in Figure 1d.

**Figure 2:**
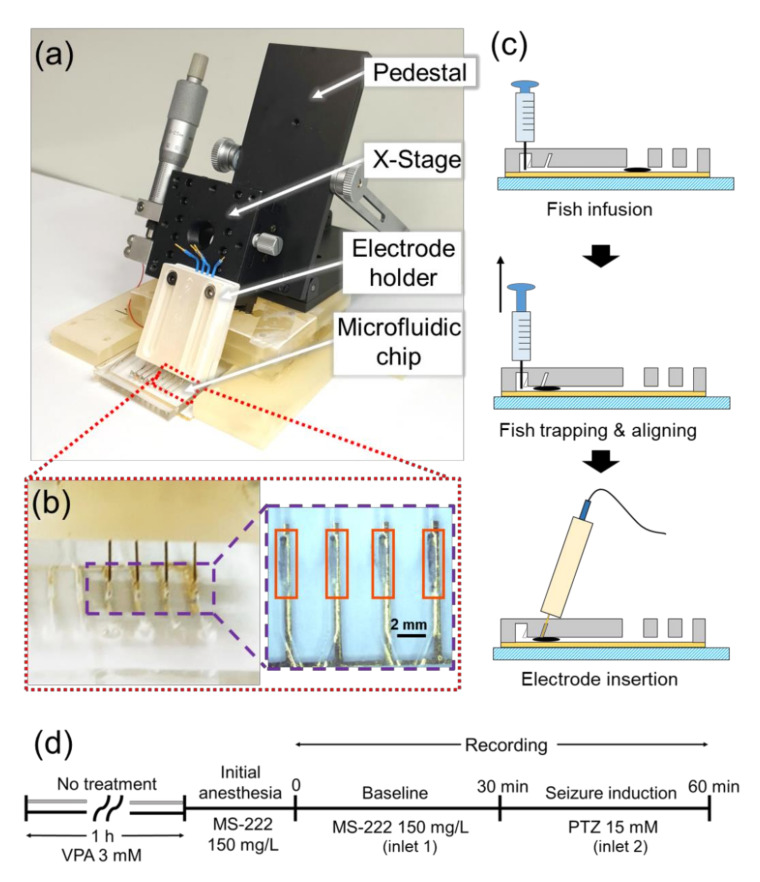
Experimental setup and protocols for electroencephalogram (EEG) recording with multiple larval zebrafish. (**a**) Whole experimental setup with a pedestal, an x-stage, an electrode holder, and a microfluidic chip. (**b**) Photograph of the needle electrodes that penetrated the PDMS membrane of the microfluidic channels containing zebrafish and magnified view of four zebrafish that were trapped and aligned in the microfluidic channels. (**c**) The way to place zebrafish (symbolized by a black ellipse) in the trapping channel by applying a negative pressure using a syringe and to insert the needle electrode toward the head of the fish. (**d**) Experimental protocols used for non-treated (gray timeline at the beginning) and valproic acid (VPA)-treated (black timeline at the beginning) group.

Figure 2a shows the entire experimental setup. The microfluidic channels could trap and align zebrafish and exchange liquids without affecting the fish inside. As shown in Figure 1b,c, there were two sets of microfluidic channels in a chip and each of them had two liquid inlets (inlet 1 and inlet 2), one fish inlet, four trapping channels, and four outlets connected to trapping channels one by one. Two liquid inlets and the fish inlet were connected in a row, and four trapping channels were spaced in parallel with an equal interval. To control the pressure in the channel as desired, an outlet was connected to a channel. In the trapping channels, guide columns were implemented to precisely guide the needle electrodes to the head of zebrafish.

The fish inlet was designed to be a dome shape with a diameter of 5 mm, in order to allow the fish to securely enter and change the direction of the head by injecting the fluid rapidly from the outlet to the inlet, creating the body rotation. All branch-shaped fluid passages were manufactured with a width of 0.6 mm and a height of 0.7 mm, but the passage between the inlets 1 and 2 had a width of 0.5 mm and a height of 0.7 mm to prevent the fish from moving backward. We chose these sizes based on the previous studies by Hong et al. (2016) and Fuad et al. (2017) [21,27]. The connecting part between the trapping channel and the outlet had a width of 0.14 mm to block the forward movement of zebrafish and a height of 1.2 mm not to block the fluid flow completely when zebrafish existed in the trapping channel. To generate a stable pressure difference for fish movement, the heights of the outlets and the guide columns were designed to be 3 and 3.5 mm, respectively, lower than the height of the liquid inlets and fish inlet of 5 mm, as shown in Figure 1c. In this way, fish could move only into the channel where a negative pressure was applied through the syringe connected to the outlet of that particular channel, while other outlets were blocked by the PDMS membrane as shown in Figure 1c, thereby no pressure difference present between those outlets and the inlets. The dimensions of the guide columns were 0.58 mm × 0.58 mm at the bottom and 0.30 mm × 0.30 mm at the top. They were inclined with an angle of 51 degrees with respect to the horizontal plane in order to target the head of fish precisely as shown in Figure 1c and Figure 2c.

### 2.2. Animals and Chemicals

Three days-post-fertilization (dpf), wild-type larval zebrafish were provided by the Zebrafish Center for Disease Modeling (ZCDM), Korea and maintained in a standard zebrafish facility at Daegu Gyeongbuk Institute of Science and Technology (DGIST) at 27 °C with 12 h: 12 h light: dark cycle for at least 2 days prior to experiments to relieve the stress caused by shipping. All experimental procedures were approved by the Institutional Animal Care and Use Committee of DGIST (approval no.: DGIST-IACUC-19052104-02, date: 21 May 2019).

As the invasive electrodes needed to precisely target the heads of tiny zebrafish, zebrafish’s movement during the placement of electrodes could cause severe damage to their body. To prevent this, we used neutralized 150 mg/L 3-aminobenzoic acid ethyl (MS-222, Merck, Darmstadt, Germany) with bicarbonate buffer solution (Merck, Darmstadt, Germany) as an anesthetic drug [32,33,34] and 15 mM PTZ (Merck, Darmstadt, Germany) [7,9,11,16,20,35] as a convulsant. To demonstrate that the developed microfluidic chip could be used for drug screening, we used 3 mM of valproic acid sodium salt (VPA) (Merck, Darmstadt, Germany) as AED, since it is most popularly used. In literature, however, the concentration and exposure time of VPA ranged variously from 200 μM to 3 mM and 1 to 24 h, respectively [10,36,37,38]. Based on preliminary tests, we decided to use 3 mM of VPA for 1 h prior to recording. A syringe containing MS-222 was connected to the inlet 1 that was located more distant to the fish inlet and another syringe containing PTZ was connected to the inlet 2 to prevent mixing with MS-222.

### 2.3. Fish Infusion and Needle Electrode Insertion

First, larval zebrafish at 5 to 7 dpf were transported to MS-222 solution using a pipette, for anesthesia. After no movement of fish was confirmed, the fish were transported into the fish inlet of the microfluidic chip through a syringe with an 18-guage needle. When the solution containing the fish filled the fish inlet, we penetrated the 1 mm-thick PDMS membrane of an outlet using a syringe with a 23-guage needle, and applied a negative pressure to the particular channel corresponding to that outlet, as described in Figure 2c. The liquid containing the fish could move only in the direction where the pressure difference existed, because the outlets of other channels were blocked by the PDMS membrane. Using the syringe, we could adjust the head direction of the fish in the dome of the fish inlet and forced the fish to move into the trapping channel. We repeated the process to position all four fish in the trapping channels. Although the trapping channels that were already occupied by the fish had the penetrated PDMS membrane, there was little pressure difference between the inlet and the outlet because the fish blocked the pathway of fluid flow (Figure 2b).

To detect EEG, tungsten needle electrodes (A-M Systems, Sequim, WA, USA) were used, of which diameter was 254 μm and tip exposure was 1 mm. We inserted needle electrodes into the microfluidic channels from outside, adapting the study by Cho et al. (2020) [24], but with an angle of 51 degrees in order to target the fish’s head precisely, as shown in Figure 2a. If the guide columns were standing vertically, the inside of the trapping channel was invisible due to severe diffraction caused by the roughness of the PDMS surface. Thus, the guide columns were designed to be inclined so that the tip of the electrode facing the head could be seen.

In Figure 2a, we attached an x-stage to an angle-adjustable pedestal and fixed the angle, so that the stage could only move in one direction. We manufactured the electrode holder to keep the needle electrodes linearly during the experiment, using a 3D printer (ProJet 3500 3D Printers, Rock Hill, SC, USA). We connected the x-stage and the electrode holder and lowered the four electrodes at the same time. When the electrodes penetrated the PDMS membrane, we kept lowering the electrodes slowly until the neural signals started to appear.

### 2.4. Recording EEG Signals

The needle electrode in contact with the head of the fish and the ground/reference electrode in contact with the belly of the fish were connected to a signal acquisition system (MP 36, Biopac System, USA). Recording was started after MS-222 was applied through the syringe pre-connected to the inlet 1, in a faraday cage with a dimension of 1700 mm × 1400 mm × 2100 mm, while the light was turned off. The sampling rate was 1 kHz and the signals were pre-filtered from 0.5 to 35 Hz. As shown in Figure 2d, two recording protocols were used for non-VPA-treated (VPA-) and VPA-treated (VPA+) groups. In the non-treated group, the baseline (PTZ-) was recorded for 30 min and then, PTZ was introduced (PTZ+) to the microfluidic channels for a minute to wash out and replace MS-222, through the syringe pre-connected to the inlet 2. After about 10 s, the EEG signal started to be recorded for 30 min during PTZ application. The step of liquid exchange was carried out very carefully so that the position of the electrodes and fish would not be affected during liquid exchange. We performed EEG recordings from 36 larval zebrafish, with 4 fish at once. 

In addition, we conducted another set of experiments to observe the effect of VPA, by pre-treating the fish for 1 h in 3 mM of VPA solution. After VPA pre-treatment, the fish were moved to MS-222 for anesthesia and EEG signals were recorded as described above. We pre-treated 8 larvae and recorded EEG signals from two sets of 4 larvae.

### 2.5. Statistical Analysis

We analyzed the recorded EEG signals using Welch’s t-test to confirm significant differences among drugs. The amplitude spectra and time-frequency plots were obtained using a symmetric Hamming window (window length n = 700) in MATLAB (MathWorks, Natick, MA, USA). The range of the frequency analyzed was 0.5~35 Hz, which was decided by a software filter built in the data acquisition system. We divided the frequency bands into delta (0.5~4 Hz), theta (4~8 Hz), alpha (8~13 Hz), and beta bands (13~30 Hz). In t-test, the significant difference between two groups was considered if *p* < 0.05. For additional statistical analysis, OriginPro 2017 (OriginLab Corporation, Northampton, MA, USA) was used.

We calculated the amount of increase in signal amplitude due to PTZ by normalizing the signal amplitude during PTZ application with the amplitude of baseline signal [39]. The amount of amplitude increase (*AI*) was calculated as:

*AI* = (*A_PTZ_* − *A_baseline_*)/*A_baseline_* × 100 (%), (1)

where *A_PTZ_* and *A_baseline_* are the averaged amplitudes after summing the amplitudes at all frequencies from 0.5 to 35 Hz of each recorded signal during PTZ application and during baseline recording, respectively. The effect of VPA was quantified by the amplitude reduction (*AR*) that was determined based on the relative increase in signal amplitude of non-treated and VPA-treated groups, as:

*AR* = (*AI_VPA_* − *AI_non_*)/*AI_non_* × 100 (%), (2)

where *AI_VPA_* and *AI_non_* are the amplitude increases caused by PTZ in the VPA-treated group and the non-treated group, respectively. In addition, the SNR was calculated by dividing the averaged amplitude of ictal-like events by that of baseline signal for around 1~2 s, which was the duration of ictal events [40].

## 3. Results and Discussion

### 3.1. EEG Recording from Multiple Larvae

Prior to recordings, we could place and align all the larvae at the desired position precisely, which was possible thanks to the optimized chip design and the way of applying negative pressure to the trapping channels one by one. Our system did not require the use of additional materials such as agarose, unlike previous studies [10,11,14,15,16,17,23]. The uniform distribution of fluids across the channels was confirmed through both simulations using finite element analysis and experiments using dyed fluids. We could exchange fluids in all four channels that contained larvae inside, with almost the same flow rate across the channels.

We successfully recorded EEG from 33 out of 44 larvae in total, including 7 out of 8 larvae of the VPA-treated group. As the result, the success rate in the EEG recording was 75% on average. This success rate was guaranteed through repeated measurements using the developed system, while previous studies only suggested a potential number of maximum zebrafish that can be recorded simultaneously [21,23] without presenting experimental data with a guaranteed rate of successful EEG recording for a single trial. We analyzed EEG from 32 larvae except one zebrafish that showed abnormal baseline signal. As the larval zebrafish were very delicate and small, they were vulnerable to the damage caused by the needle electrodes. If the electrodes can move individually and more precisely, the success rate would be improved, and it would be possible to record EEG using more than 4 fish at once. It took 70 min per recording session, including 10 min for preparation and 60 min for recording. Typically, electrophysiological analysis for zebrafish employed short-time recordings around 10 min [23] and most behavioral observations using PTZ exposure were usually performed for less than 30 min [10,37,41,42]. Although some studies using Scn1ab mutant recorded EEG signals for more than 4 h [21,23], most studies using PTZ-induced models presented recordings up to around 60 min maximum [9,11,20]. It was presumed that if zebrafish were forced to undergo seizure induction by PTZ, they might be under more stress than mutant models, and thus, less resistant during EEG recording. For these reasons, the developed EEG system and the used protocol were suitable for drug screening using PTZ-induced models as they could measure EEG for 60 min and more if necessary, and clear changes in EEG signals could be detected and quantified during this 60 min.

We consumed less than 1 mL of PTZ, 3 mL of VPA, and 5 mL of MS-222 for four fish. Especially, PTZ was used not only to induce seizure but also to wash out the previously applied MS-222. The used amount of PTZ was sufficient to wash out and replace MS-222 inside the channel. It was demonstrated that detecting EEG from multiple larvae simultaneously could reduce time and cost significantly in screening the effects of different drugs. The microfluidic chip was reusable without leakage, up to 5 trials. However, the developed system had a limitation that it was hard to assure if the fish were alive after the recording because it was difficult to remove them out of the microfluidic channels, without damaging them.

**Figure 3:**
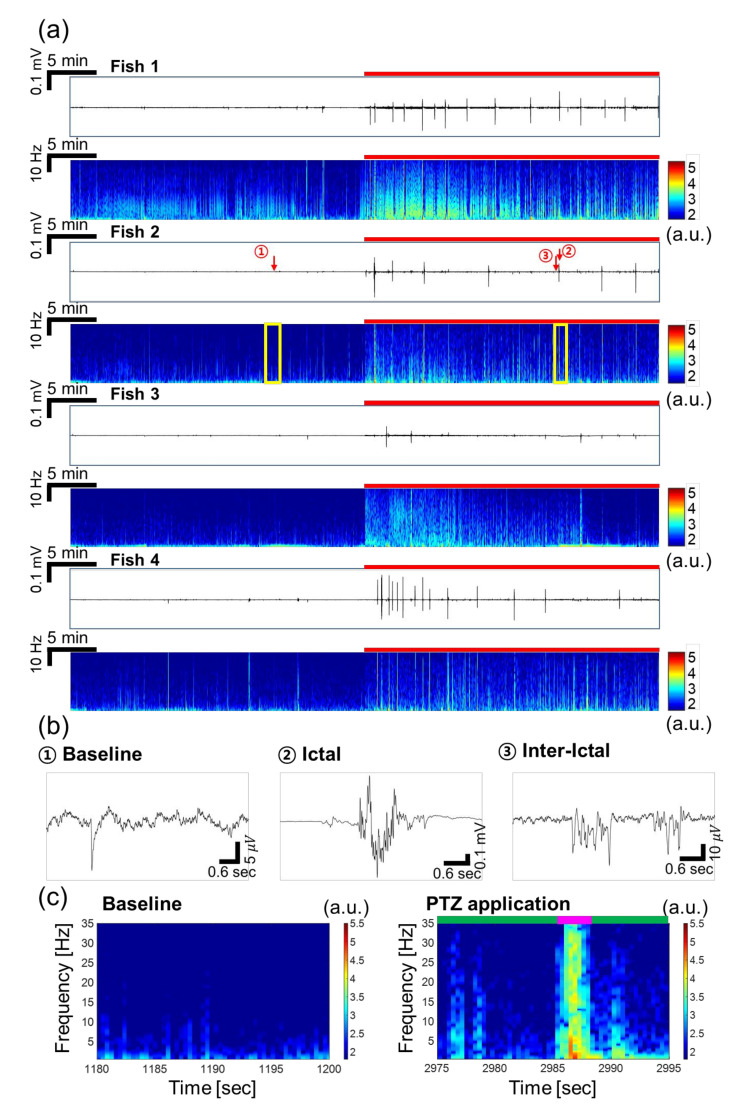
EEG signals recorded simultaneously from 4 larval zebrafish in the non-VPA-treated group. (**a**) Recorded raw data in time domain and their time-frequency plots (0.5–35 Hz). The red bar represents the duration of PTZ application. (**b**) Characteristic waveforms during baseline and PTZ application: ictal and inter-ictal events. Note that the scales for *y*-axis are all different for baseline, ictal and inter-ictal signals. (**c**) Magnified views of the time-frequency plot marked by yellow boxes in (**a**). The signal amplitude increased over a wide range of frequency when PTZ was applied, especially during the ictal event. The ictal event was represented by pink bar and inter-ictal events were represented by green bar.

Figure 3a presents EEG signals detected from four fish at once, in which the difference between the signals during baseline recording and PTZ application was clearly observed. After applying PTZ, seizure started with higher amplitudes than the baseline signal. In addition, the principal components of seizure such as ictal and inter-ictal events, as reported in Aydin et al. (2015), Acharya et al. (2014), and Fisher et al. (2014), started to be observed, as illustrated in Figure 3b [6,40,43]. The ictal signals had peak-to-peak amplitudes of around 800 μV to 1 mV with durations of about 1~2 s. For instance, the ictal events of fish 2 showed 160 times higher amplitude on average compared to the amplitude of baseline signal. The inter-ictal signal, which occurred between two ictal events, was also distinctive from the baseline signal in that oscillating signals with amplitudes of tens of microvolts were observed, as shown in Figure 3b. When comparing ictal and inter-ictal events, the peak-to-peak amplitude of ictal signals was 30 times greater than that of inter-ictal signals. The fact that we could detect these principal components of seizure after PTZ filled the channels, replacing MS-222, indicates that the electrodes were secured in desired position even after liquid exchange. The time-frequency plots in Figure 3a clearly show the increase in signal amplitude caused by PTZ, especially during the ictal events. In Figure 3c, the baseline signal showed higher amplitudes in the low frequency range below 5 Hz. On the other hand, when PTZ was applied, the signal amplitude increased during ictal and inter-ictal events significantly. During inter-ictal events, the slight amplitude increase was observed at frequencies up to near 20 Hz. During ictal events, the amplitude clearly increased in the entire frequency range up to 35 Hz. Especially, the amplitude at frequencies lower than 5 Hz was almost doubled compared to the baseline, showing that the dominant signal during ictal events was observed at this low frequency. This is coincident with the EEG signals recorded from the mutant model using non-invasive electrodes in a microfluidic channel [30].

The SNR in our invasive EEG recording was calculated to be 162 for fish 2 in Figure 3a, which was more than 13 times of those obtained using non-invasive methods. From previous studies, the SNRs using non-invasive methods were estimated to be about 10 using a flexible printed circuit board (FPCB) electrode in adults [39], 7.5 using larvae with a mesh electrode [20], and 8 to 12 using a surface electrode from larvae [21]. The SNR was greatly improved by using the invasive method, as we could eliminate the artifacts caused by movements of gills, eyes, or tails.

### 3.2. Continuous Drug Screening

**Figure 4:**
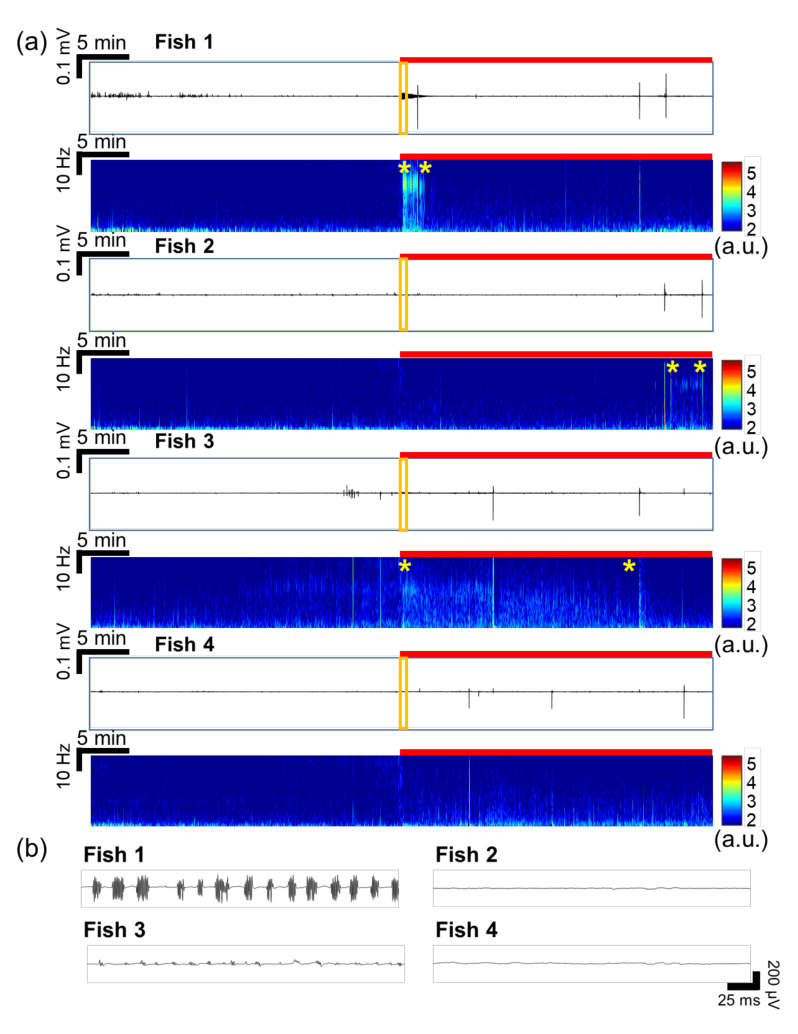
EEG signals recorded simultaneously from 4 larval zebrafish in the VPA-treated group. (**a**) Recorded raw data in time domain and their time-frequency plots (0.5–35 Hz). The red bar represents the duration of PTZ application. The intervals marked by two yellow stars show inter-ictal events. (**b**) Magnified views of the signals marked by the orange boxes in (**a**). In fish 1 and 3, inter-itcal signals were observed as soon as PTZ was applied, regardless of VPA treatment. In all fish, ictal events occurred.

**Figure 5:**
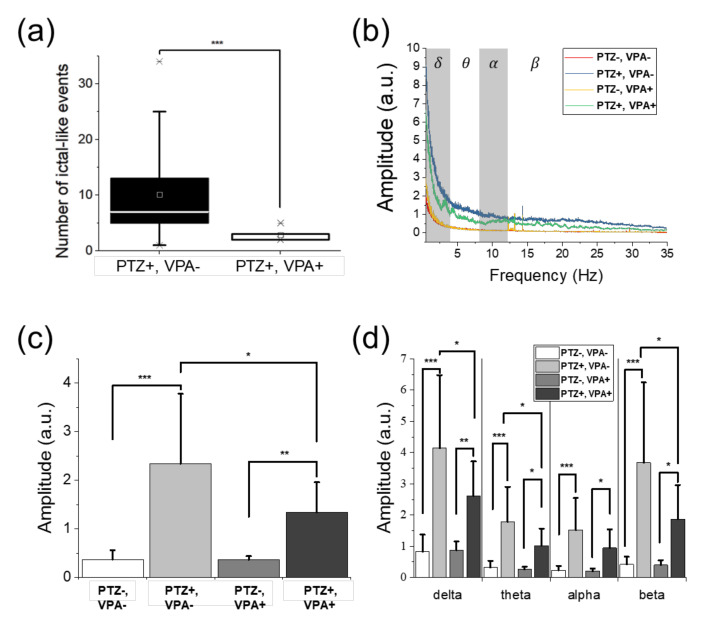
Comparison of the number of ictal-like events and the signal amplitude according to drug applications. (**a**) The number of ictal-like events in both non-treated (n = 26) and VPA-treated (n = 7) groups. In the VPA-treated group, the number of ictal-like events was reduced significantly compared to that of the non-treated group. The boxes show the range from 25% to 75% of the measured data. The lines and squares in the boxes represent the medians and means, respectively, while asterisks represent the values corresponding to 1% and 99% of the data. The error bars indicate the upper and lower boundaries of outlier. (**b**) The averaged amplitude spectra of the non-treated (PTZ-, VPA-), non-treated group with PTZ application (PTZ+, VPA-), VPA-treated (PTZ-, VPA+), and VPA-treated with PTZ application (PTZ+, VPA+) groups. The relative change in signal amplitude of each groups (**c**) over the entire frequency range and (**d**) in the divided frequency bands into delta, theta, alpha, and beta bands. In (**b**) and (**c**), the error bars indicate the standard deviations. (**a**) and (**c**) suggest that VPA treatment clearly suppressed the amplitude increase by PTZ. Significant differences were denoted as * for *p* < 0.05, ** for *p* < 0.01, and *** for *p* < 0.001.

Figure 4a shows the recorded signals from 4 fish at once, in which the fish were pre-treated by VPA (VPA-treated group). In Figure 4a, the number of ictal events and high-amplitude signals significantly decreased with VPA treatment, compared to the EEG signals of the non-treated group in Figure 3a. In Figure 4a, there were no significant differences between the signals during baseline recording and PTZ application in the time-frequency plots except some intervals during ictal events as marked by yellow stars. On the other hand, inter-ictal signals were observed in fish 1 and fish 3, as shown in Figure 4b. Particularly, fish 1 responded a lot upon the initial application of PTZ for 2~3 min. Based on these observations, it was concluded that seizure was clearly suppressed by VPA treatment. As presented in the time-domain signals of Figure 3a and Figure 4a, the number of ictal-like events including ictal events decreased when VPA was treated. In Figure 5a, the average number of ictal-like events was 10.12 in the non-treated group and 2.85 in the VPA-treated group, indicating that ictal-like events were suppressed by VPA. The maximum number was 34 and 5 and the minimum was 1 and 2 in the non-treated group and VPA-treated group, respectively.

Signals in time domain as well as frequency domain ranging from 0.5 to 35 Hz showed a variation in amplitude caused by PTZ and VPA, as described in Figure 5b. As shown in Figure 5c and Table 1, the signal amplitude increased when PTZ was applied regardless of VPA treatment. The EEG signal in the VPA-treated group showed smaller amplitude compared to that in the non-treated group during PTZ application, indicating the brain activities were suppressed by VPA. The signal amplitude increased by 534% when PTZ was applied in the non-treated group and by 272% in the VPA-treated group, as shown in Figure 5c. As the result, VPA treatment suppressed the brain activities increased due to PTZ, by 49% when comparing the non-treated and VPA-treated groups. It was also reported from behavioral observations, in which drastic movements and increases in moving distance were observed when PTZ was applied, and the movements were suppressed when VPA was applied [10,37,41].

Figure 5b suggests that the EEG signals had the dominant amplitude in the frequency range lower than 5 Hz. Figure 5d and Table 1 describe the changes in signal amplitude according to the frequency bands. The delta and beta bands were the most dominant bands with higher amplitudes. The delta band showed the largest amplitude during the baseline recording of both the non-treated and VPA-treated groups. It may be because zebrafish were sleeping due to MS-222 during the experiment [44,45]. On the other hand, the amplitude in beta band increased by 776% and 363% in the non-treated and VPA-treated groups, respectively, when PTZ was applied. From the delta band to the beta band, the higher frequency band showed the larger increase in signal amplitude by PTZ. On the other hand, the amounts of signal reduction by VPA in the delta and beta bands were similar, by 50% and 53%, respectively, as shown in Table 1. The highest reduction rate was observed at the delta and beta bands, which was different from the suppression effects observed in adult zebrafish with higher reduction rates at lower frequencies consistently [39]. Both the results from larvae and adults, however, indicate that VPA suppressed the delta band most significantly (reduction rate ≥ 50%). Based on this result, it can be confirmed that the delta band is correlated with seizure, in agreement with previous studies [39,46,47,48], although the delta band is related to sleeping. Overall, VPA suppressed the amplitude increase due to PTZ by suppressing the EEG amplitudes over the entire frequency range.

## 4. Conclusions

We developed a microfluidic EEG system that can detect EEG invasively from multiple larval zebrafish simultaneously to obtain high-SNR signals, by devising a microfluidic chip to trap larval zebrafish stably and exchange the liquid uniformly across all channels. Through the branch-shaped microfluidic channels, the liquid could be filled evenly with the same gradient across the channels, even with the larvae contained inside. Using the invasive EEG detection, EEG recordings were free from displacement or detachment of the electrodes even during liquid exchange. The developed microfluidic chip achieved stable fixation of fish during the EEG recording, without using any additional means such as agarose, unlike previous studies. In addition, we could expose the fish to multiple drugs sequentially during EEG recording without any disturbances that could affect the EEG signal. We recorded EEG signals from 4 fish at once for 60 min, which decreased the experimental time and drug consumption to a quarter per fish. A success rate of 75% was achieved in the EEG recording. However, if the movement of electrodes can be adjusted in a finer way, the number of measurable fish and the success rate would be improved, as the safe and reliable trapping of fish was guaranteed using the developed microfluidic chip. The small size of larval zebrafish and the use of microfluidic channels enabled the sequential drug screening while the fish stayed in a stable aquatic environment. Additionally, the responses to drugs could be immediately observed because drugs were directly exposed to the fish owing to the continuous flow through the developed microfluidic chip, unlike the cases using agarose to fix the larvae. By observing changes in EEG signals, we could successfully demonstrate the effects of convulsant and AED quantitatively and statistically. The developed EEG system is expected to improve the efficiency in EEG detection greatly for drug screening to treat neurological disorders such as epilepsy, thanks to its small size, low manufacturing cost, and the guaranteed uniform drug infusion across all channels with no environmentally induced artifacts.

## Figures and Tables

**Table 1:** Amplitude increase by PTZ in both non-treated and VPA-treated groups, according to frequency bands, and resultant amplitude reduction by VPA pre-treatment.

EEG Band	Amplitude Increase (%)		Reduction (%)
	Non-Treated	VPA-Treated	
Delta (0.5~4 Hz)	403	202	−50
Theta (4~8 Hz)	462	294	−36
Alpha (8~13 Hz)	559	367	−34
Beta (13~30 Hz)	776	363	−53
Total (0.5~35 Hz)	534	272	−49
